# Antibiotic resistance and mitigation using One Health lens in aquaculture of Northern Nigeria

**DOI:** 10.4102/ojvr.v91i2.2165

**Published:** 2024-10-16

**Authors:** Nma B. Alhaji, Ismail Ayoade Odetokun, Mohammed S. Jibrin, Mohammed K. Lawan, Jacob Kwaga

**Affiliations:** 1 Department of Epidemiology/Preventive Veterinary Medicine, Faculty of Veterinary Public Health and Preventive Medicine, Postgraduate College of Veterinary Surgeons, Abuja, Nigeria; 2 Department of Food Safety, Africa Centre of Excellence for Mycotoxin and Food Safety, Federal University of Technology, Minna, Nigeria; 3 Department of Veterinary Public Health, Faculty of Veterinary Medicine, University of Ilorin, Ilorin, Nigeria; 4 Department of Veterinary Public Health, Veterinary Teaching Hospital, Usmanu Danfodiyo University, Sokoto, Nigeria; 5 Department of Veterinary Public Health, Faculty of Veterinary Medicine, Ahmadu Bello University, Zaria, Nigeria

**Keywords:** antibiotic usage, antibiotic residue, antibiotic resistance, freshwater aquaculture, fish, One Health

## Abstract

A cross-sectional study was conducted to assess antibiotic usage, residues, resistance and drivers of their emergence in fish aquaculture in Northern Nigeria between 2019 and 2020. A structured questionnaire was administered to 151 randomly selected farmers. Fish, column and wastewater samples were analysed with enzyme link immunosorbent assay (ELISA) for residue detection and quantification. We performed descriptive and analytic statistical analyses. All selected farmers participated, 78.1% used antibiotics in ponds. Majority (77.1%) of the farmers did not know what antibiotic misuse entailed and 73.3% mentioned that antibiotic misuse and overuse cannot predispose to residues and resistance. The most frequently used antibiotics were tetracyclines (TCs) (99.2%). Significant risk routes for residue and resistance spread were: contaminated fish and its products ingestion, direct contact with fish and fomites contaminated as well as water and aerosols contaminated in the pond environment. Significant social and cultural that influenced residue and resistance development include antibiotics misuse/overuse (Odds Ratio [OR] = 3.8; 95% confidence interval [CI]: 1.62–8.74) as well as poor education and expertise of the farmers (OR = 2.9; 95% CI: 1.24–6.94). The mean TCs residues level in column and wastewater was 123.6 ± 18.2 μg/kg^−1^. Poor knowledge and attitudes regarding antibiotic usage were identified. Adequate antibiotic stewardship should be promoted through farmers’ education. Identified social and cultural factors can be mitigated through the ‘One Health’ approach.

## Introduction

Of the total world aquatic animal production, aquaculture produced 76.6 million tonnes (45%) of aquatic animals and more than 53% of the global fish consumption in 2015 (FAO 2017). Fish contributes 17% of global animal proteins needed for human nutrition (Troell et al. 2014). Per capita food of fish origin consumption rose from 19.7 kg to 20.3 kg between 2013 and 2015 (FAO 2017). Globally, aquaculture contributes about 10% of the total animal proteins for humans and it may increase up to 50% by 2030 (World Bank 2013). However, these positive contributions are being undermined by the effects of misuse and overuse of antimicrobials (Alhaji, Maikai & Kwaga 2021).

The intensification and semi-intensification of aquaculture create conditions that promote infectious disease occurrence, disease-related issues and biofouling because of poor biosecurity, sanitation and hygiene (Santos & Ramos 2019). Consequently, antibiotic agents have been used prophylactically, therapeutically and metaphylactically to control and prevent diseases, and for promotion of growth (Carvalho & Santos 2016; Okocha, Olatoye & Adedeji 2018). They are often administered to fish via their feed, by injections and in baths (Chowdhury et al. 2022). Antibiotic use in food animals, including aquaculture, was estimated globally at 63 151 tonnes in 2010 with an expected increment of 67% by 2030 (Van Boeckel et al. 2015). However, antimicrobials have not always been used appropriately in aquaculture environments (FAO/WHO 2003).

The use of antibiotics in aquaculture has not been regulated in many low-income countries (LICs), with resultant misuse and overuse that consequently exert pressure on pathogens and antimicrobial resistance (AMR) development, an emerging global health threat to humans and animals (FAO 2016). Antibiotic misuse and overuse in aquaculture have been specifically associated with the presence of antimicrobial residues and resistance development resulting from excretion into the surrounding freshwater and marine environments either as bioactive metabolites or as original compounds (Hatosy & Martiny 2015; Kuemmerer 2009; Nakayama et al. 2017; Rico et al. 2014). It was estimated that out of 92 700 tonnes of antibiotics used in 2013, an estimated 53 800 tonnes were excreted into the environment (Zhang et al. 2015). Occurring pollutants that presently attract worldwide attention are allergic reactions in humans, antibiotic residuals and resistant pathogens and genes (Bousquet 2009; Pazda et al. 2019; Rizzo et al. 2013).

The most commonly used antibiotics for the treatment and prevention of infections in animals are excreted out of their bodies via faeces and urine as wastes, which contaminate both terrestrial and aquatic environments and cannot be eliminated from the latter because most of them are not biodegradable (Baran et al. 2011; Miranda et al. 2013; Serrano et al. 2021). Antibiotic residues wield intense pressure on environmental microbiota leading to the genetic selection of resistant bacteria as well as exerting potentially long-term adverse effects on human and animal health (Ahmed et al. 2015, 2017; Zhao et al. 2016).

Antibiotic residues and resistance in aquaculture environments are considered a One Health challenge, both as cause and solution (Bhushan et al. 2017). Discussions at the human-animal-environment interface can provide solutions for the control and prevention of usage and resistance of antibiotics locally and globally (Ogawa et al. 2019; Sanderson et al. 2019). As part of global efforts to mitigate AMR, the World Health Organization’s (WHOs) Global Action Plan on AMR addresses the need to bridge the knowledge gaps and evidence-based practices of antibiotic use through surveillance and research (WHO 2017). Although the aforementioned are key facets of intervention strategies, they are challenged by poor biosecurity, sanitation and hygiene as well as inadequate diagnostics in farms in LICs (Queenan, Häsler & Rushton 2016). Strengthening surveillance and research on antibiotic stewardship in freshwater fish aquaculture will immensely contribute to the mitigation of AMR arising from the food chain of fish origin (Collineau et al. 2016).

As in other food-producing animals, AMR mitigation in freshwater fish aquaculture farms will require farmers’ adequate knowledge and attitude on the appropriate use of antibiotics, resistance emergence and factors that drive it, especially in countries where the growth of the industry and disease risk are increasing (Alhaji et al. 2018).

The objectives of this study were to assess the knowledge and attitudes of the farmers towards the use of antibiotics and pathways for the residues and resistance spread from freshwater aquaculture. We also determined the presence and quantities of residues in tissues and hypothesised that socio-economic activities can’t drive the occurrence of antibiotic residue and resistance in freshwater fish aquaculture establishments in Nigeria. Furthermore, we designed a One Health conceptual framework towards antibiotic resistance (ABR) intervention in freshwater aquaculture. The results of this research will provide background information for policymakers on antibiotic stewardship, surveillance and food safety in freshwater farming in developing economies.

## Materials and methods

### Study area, design, and population

The study was conducted in 151 freshwater fish aquacultures located in 63 local government areas of the Southern Guinea Savannah ecological zone of Nigeria. The zone has three sub-aqua-ecological zones characterised by variable climatic conditions (alternating warm and cold temperatures, favourable humidity and long duration of rainfall suitable for freshwater fish farming), many water bodies, rivers, streams, *fadamas* and dams (www.nigerstate.gov.ng).

A mixed study design of a cross-sectional study and participatory epidemiology (PE) approach was carried out between 2019 and 2020, and targeted freshwater fish aquaculture farmers and their ponds domiciled in rural and urban areas. For this research, participants were eligible for selection if each fulfilled the condition of being an artificial or natural pond fish farmer culturing fish for sustainable economic livelihood. Each participant has to engage in either an intensive management system, in which fishes are fed with an external food supply, or an extensive system that involves feeding fishes with phytoplankton and zooplankton naturally available, with no supplementation diets.

### Determination of sample size

Sample size determination was performed with the OpenEpi version 3.1 software (Dean, Sullivan & Soe 2013). The power was set to 50% at 95% confidence level and 8% degree of precision for the study area (Southern Guinea Savannah zone). A total of 151 fish farms were obtained as a sample size and enrolled in the study. Samples were collected from 151 fish farms across the study area. Also, the sample size for biological samples was obtained using 50% power and an 8% margin of error set at 95% confidence level. The 151 biological samples were made up of fish tissues (fillets and livers, which are bioaccumulate residues), and pond water was collected and used for antibiotics screening.

For the participatory survey, preliminary information on both intensive and extensive fish farming communities was obtained from the Department of Fisheries in the region. These communities, domiciled in the three sub-aqua ecological zones, were identified and 9 out of the total 15 were conveniently considered. However, the number of communities considered per ecological zone for the PE exercises depended on the numbers and sizes of the available clusters of fish farms (concentration of two or more fish farming households in a settlement). Nine fishing communities were purposively selected. Two key informants were conveniently considered in each of the chosen fish farming communities, and a total of 18 key informants were considered and led by other fish farmers who participated in the PE exercises. However, the number of other participants was unrestricted.

### Sampling methods

A multi-stage sampling procedure was conducted to select the aquaculture ponds. In the first stage, the whole Southern Guinea Ecological zone was conveniently considered for sampling. For the second stage, the three sub-aqua-ecological zones were purposively considered, and fish pond registers of the three sub-zones were obtained from the Zonal Fisheries Offices as sampling frames. In the third and final stage, a systematic random sampling procedure, with a sampling interval of one, was conducted for pond selection. A sampling interval was obtained by dividing the calculated sample size (*n* = 151) by the number of biological samples (fillet, liver and pond water) expected to be collected (*n* = 151). A minimum of 50 freshwater farms were selected in each aquaculture sub-ecological zone, and biological samples were taken from them for quantitative antibiotic residue screening.

For the PE survey, key criteria for the selection of the fish farming communities were based on the number of available fish farm clusters in each zone as mentioned earlier. A purposive sampling method was used to select the communities and key informants for PE exercises

### Questionnaire tool

We designed a structured questionnaire that contained close-ended questions to ease response precision and used it to collect quantitative data (Thrusfield 2009). Questions were based mostly on literature and experts’ opinions. The tool had four sections: (1) socio-demographic characteristics of respondents (ponds owners or managers); (2) aquaculture ponds information; (3) knowledge about antibiotic usage in fish ponds, pathways for antibiotic residues and resistance spread to humans, and the socio-economic variables that drive residues and resistance occurrence; and (iv) attitudes towards antibiotic usage in freshwater fish aquaculture ponds. Six animal health technologists were recruited, trained and performed the interviewer-administered questionnaire exercise. The questionnaire was pre-tested on five farmers in the southern aquaculture-ecological zone and all gaps in the variables were identified and corrected to ease subsequent adequate data delivery. For quality control, we supervised questionnaire administration daily.

We verbally obtained the informed consent of participants before the commencement of questionnaire administering exercises as well as verbally informed of the survey aim. Respondents were informed of responses’ confidentiality, voluntary participation and the opportunity to withdraw at will without prejudice.

### Samples collection

We considered the fact that most antibiotics administered to fishes are excreted into the pond water column unchanged and pass to water bodies as aquaculture discharges and run-offs, with major residues and resistance potentially impacting the environment. Therefore, fish, column and wastewater samples (15 fillets, 15 livers and 121 water samples) were collected from the 151 ponds and a few surrounding water bodies. Approximately, each tissue (fillet or liver) weighed 1 g and the water sample weighed 4 mL. We aseptically collected the samples in sterile sample bottles, packed them in an icebox at the temperature of 4 °C and took them to the laboratory for antimicrobial residue screening.

### Competitive enzyme link immunosorbent assay

Each fish’s fillet or liver sample was triturated with a mixer, a 2 g homogenised sample was weighed in a 50 mL centrifuge tube and 4 mL 1% solution of trichloroacetic acid (Liquor 1) was added. This was oscillated for 2 min and centrifuged at 4000 rpm (revolutions per minute) at 37 °C for 10 min. About 250 μL supernatant was wiped out and 750 μL redissolving solution (Liquor 2) was added to dissolve the dried residue, and 50 μL of each sample was used for the assay. (Dilution factor: 8; detection limits: 0.4 parts per billion [ppb]). Also, about 1900 μL redissolving solution was added to each pond water sample to dilute and was mixed for 30 s; 50 μL of each sample was used for the assay. (Dilution factor: 40; detection limits: 2 ppb).

A tetracyclines (TCs) kit (Solarbio^®^ Life Sciences) was used for the determination of the TCs residues in samples. The ELISA kit was used for quantitative analysis of TC in tissues and conducted following the manufacturer’s guidelines. A calibration curve was plotted between the absorbance percentages of standards on the *y*-axis and the corresponding log of standards concentration (ppb) *on* the *x*-axis. A standard semi-log curve was drawn; the absorbance percentages were substituted and the corresponding concentrations were obtained from the standard curve. This was multiplied by the corresponding dilution factor to obtain the actual concentration of TCs in the samples.

### Participatory data collection

The PE exercises were conducted using the participatory tools as described (Catley, Alders & Wood 2012; Alhaji & Babalobi 2016). We used participatory rural appraisal (PRA) tools of key informants, semi-structured interviews (SSI), checklists, proportional piling and triangulation to collect qualitative and semi-qualitative data.

### Key informants, semi-structured interviews and checklist

A key informant was an elderly farmer with equal to or more than 10 years of fish farming management experience and commands much respect from members of the same occupation in the fishing community. In the participatory appraisal, only a SSI was used. The study team used a ‘checklist’ of important points covered during SSI, which were basically on antibiotics used and reasons for usage on ponds. It made the interview flexible and allowed respondents to express their words within their conceptual thoughts. The introduction of the appraisal team, as well as the aim, were done to the participants before commencing each PE session.

We conducted an SSI session for about an hour in each community. To facilitate discussion, the appraisal team firstly asked questions about fish ponds’ general management, secondly followed by specific questions on important antibiotics used in the management of bacterial infections, and thirdly other antibiotic uses in ponds within the preceding 1–5-year period of the interview. A checklist of open-ended questions was used to guide and standardise discussions, while the key informants’ responses were probed.

### Proportional piling of frequently used antibiotics

Counters (pebbles), flipcharts and permanent markers were used for this exercise. Farmers and the appraisal team compiled a list of commonly used antimicrobials mentioned during SSI. Farmers drew circles on the flip charts, each representing mentioned most commonly used antimicrobial. Some 100 pebbles were given to them and told to be piled in the respective named antibiotic circles proportionally to the frequency of perceived usefulness on the ponds. After consensus was reached among them, pebbles in each circle were counted to give a proportion that represented the relative frequency of each commonly used antimicrobial on the ponds in that particular fish farming community.

### Triangulation and key biological sampling

At the end of each proportional piling exercise in a community, participants cross-checked and debated on the piles until an agreement was reached on the obtained data. At the end of all the PE exercises in the nine (*n* = 9) fish farming communities, the appraisal team further triangulated (compared) obtained results and average scores or piles were computed. They represented mean final semi-quantitative (participatory) outcomes of the perceived relative frequency of antibiotics used in the ponds. Also, the appraisal team used the results of antibiotic screening (key biological samples) to triangulate or validate the semi-quantitative outcomes obtained during piling exercises (Mariner & Paskin 2000).

### One Health conceptual framework

**FIGURE 1:**
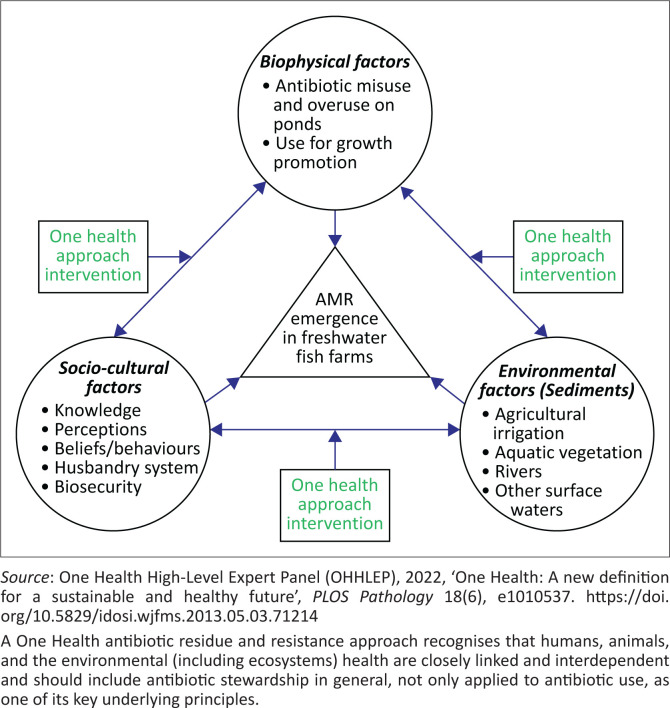
A One Health conceptual framework for antibiotic residue and resistance emergence and intervention points in freshwater fish farms.

The One Health conceptual framework was designed to assess interactions of the various biophysical factors, environmental exigencies and the sociocultural activities that interface to drive antibiotic residues and resistance emergence in aquaculture. The model illustrated the interactions of the domains that converged to drive the emergence and points for One Health interventions (Figure 1).

### Data management and statistical analysis

We entered generated data into Microsoft Excel 7 (Microsoft Corporation, Redmond, Washington, United States [US]) spreadsheets, and analysed with OpenEpi version 3.1 (Dean et al. 2013). Descriptive statistics was used for response variables on knowledge and practices on antimicrobial use and resistance was presented as frequencies and proportions.

To assess associations of social and cultural factors (independent or explanatory variables) with antibiotic residue and resistance occurrence in the fish ponds, we created dependent (outcome) variables from the participant’s responses during interviewer-questionnaire administering. A 0–9 rating score scale, which represented response stringency to questions, was designed. We considered response score values within the range of 0–4 points as ‘poor’, and those within 5–9 points as ‘satisfactory’. Univariate analysis was first performed to assess associations between the explanatory and outcome variables using Chi-square tests or Fischer’s exact tests, where necessary (Dohoo, Martin & Studahl 2012). Variables found to be significant were then modelled using a likelihood backward multivariable logistic regression to control for confounders and effect modifiers. The difference between antimicrobial residue concentrations in the samples was determined with analyses of variance (ANOVA). A *p*-value set at 0.05 was considered significant in the analyses.

We used Kendall’s Coefficient of Concordance W statistic, a non-parametric statistic (Kendall & Smith 1939; Legendre 2010; Siegel & Castellan 1988), to analyse the semi-quantitative data obtained from the PE exercises for the level of agreements among the farmers in the fish farming communities. *W* values vary from 0 to 1. *W* = 0 indicates there was no agreement and *W* = 1 means there was very strong agreement at 95% confidence level. A *p* < 0.05 was considered statistically significant for the agreement among them. Furthermore, we used descriptive statistics, mean and proportions (%) to analyse data on different frequently used antibiotics in the fishing communities. An online software programme (https://www.statstodo.com/KendallW.php) was used.

### Ethical considerations

The study proposal was submitted and cleared by the Niger State Ministry of Livestock and Fisheries Research Ethics Committee Ref. MLF/NGS/REC/03/2017. *Clarias gariepinus*, also known as African catfish, were sampled and humanely sacrificed (Blessing, Marshall & Balcombe 2010).

## Results

### Participants’ socio-demographic characteristics

**FIGURE 2:**
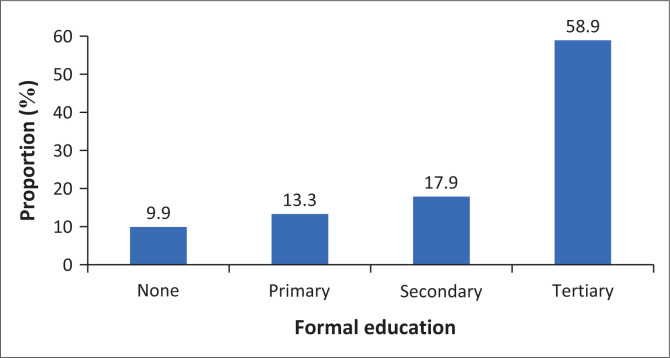
Formal educational status of aquaculture fish farmers in the Southern Guinea Ecological Zone of Northern Nigeria.

All the selected 151 aquaculture pond farmers responded and 78.2% (*n* = 118/151) indicated antibiotic use in fish farms. About 88.7% (*n* = 134/151) of the participants were males and 85.4% (*n* = 129/151) of them were married. Furthermore, 58.9% (*n* = 89/151) of the participants possessed tertiary education, whereas 9.9% (*n* = 15/151) of them had no formal education (Figure 2).

### Knowledge regarding the use of antibiotics in freshwater fish farms

About 78.1% (*n* = 118/151) of the participants reported possessing knowledge about antibiotic usage in freshwater fish aquaculture, while 21.9% (*n* = 33/151) did not. Those with the knowledge obtained index information from friends (51.7%, *n* = 61/118), relations (40.7%, *n* = 48/118) and animal health personnel (7.6%, *n* = 9/118). Few farmers (22.9%, *n* = 29/118) knew antibiotics for being used in the treatment of infections in freshwater aquaculture and 49.2% (*n* = 58/118) reported prevention. Furthermore, 22.0% (*n* = 26/118) of the farmers knew the drugs to be used for metaphylaxis. Also, 11.0% (*n* = 13/118) responded knowing misuse of antibiotics in freshwater fish aquaculture when given without prescription, 7.6% (*n* = 9/118) knew it to be when administered for viral and fungal infections, and 77.1% (*n* = 91/118) did not know about antibiotic misuse. However, 26.3% (*n* = 31/118) agreed that antibiotic misuse and overuse in freshwater aquaculture predisposed to residue and resistance emergence, while 73.3% (*n* = 87/118) of them disagreed. In addition, 41.5% (*n* = 49/118) did not know about the effects of residue and resistance in aquaculture fish (Table 1).

**TABLE 1:** Fish farmers’ knowledge regarding the use of antibiotics in freshwater fish aquaculture in the Southern Guinea Ecological Zone of Northern Nigeria.

Variable	Frequency (*n*)	Proportion (%)	95% CI
**Known antibiotics to be used**			
To treat infections in fishes	27	22.9	15.98, 31.10
To prevent infections in fishes	58	49.2	40.21, 58.14
To promote growth in fishes	7	5.9	2.63, 11.38
All of the above	26	22.0	15.25, 30.18
**Antibiotic misuse is when**			
Administered without prescription	13	11.0	6.27, 17.66
Administered for viral and fungal infections	9	7.6	3.78, 13.53
Administered for growth promotion	5	4.2	1.57, 9.14
Don’t know	91	77.1	68.90, 84.02
**Misuse and overuse of antibiotics can predispose to residue and resistance emergence in fish**			
Agree	31	26.3	18.93, 34.76
Disagree	87	73.7	65.24, 81.07
**Antibiotic residue and resistance effects in fishes**			
Non-response to infection treatment	27	22.9	15.98, 31.10
Extra costs on infection treatment	42	35.6	27.35, 44.54
Don’t know	49	41.5	32.89, 507.57
**Antibiotic residue in fish aquaculture spread to humans**			
Agree	20	16.9	10.97, 24.53
Disagree	98	83.1	75.47, 89.03

CI, confidence interval.

### Attitudes towards antibiotic usage in freshwater fish aquaculture

About 78.0% (*n* = 118/151) of the farmers used antibiotics in freshwater fish aquaculture. Meanwhile, 94.1% (*n* = 111/118) of farmers practised self-prescription of antibiotics used, while 5.9% (*n* = 7/118) patronised veterinarians’ services. About 62.7% (*n* = 74/118) of the farmers acquired antibiotics used in ponds from veterinary pharmacies, 26.3% (*n* = 31/118) from human pharmacies and 11.0% (*n* = 13/118) patronised drug hawkers. Also, 56.8% (*n* = 67/118) of the farmers carried out arbitrary application of antibiotics in ponds, and 43.2% (*n* = 51/118) used accompanied label instructions. Furthermore, 94.9% (*n* = 112/118) of the farmers conducted self-administration of antibiotics used in fish aquaculture, while 5.1% (*n* = 6/118) invited the services of veterinary authorities.

On the rate of antibiotics use on the farms, 20.3% (*n* = 24/118) of the farmers used prescribed leaflet instructions, a single high dose of antibiotics was daily administered by 26.3% (*n* = 31/118) of the farmers, while a single high dose of antibiotics was weekly administered by 53.3% (*n* = 63/118) of them. However, only 5.1% (*n* = 6/118) of them observed antibiotic withdrawal periods in fish aquaculture after usage while 94.9% (*n* = 116/118) did not observe it. While 7.6% (*n* = 9/118) of the farmers applied antibiotics for growth promotion, 34.7% (*n* = 41/118) of them used antibiotics for metaphylaxis (Table 2).

**TABLE 2:** Farmers’ attitudes towards antibiotic usage in freshwater fish aquaculture in the Southern Guinea Ecological Zone of Northern Nigeria.

Attitude	Frequency (*n*)	Proportion (%)	95% CI
**Prescription of antibiotics used in aquaculture**			
Self-prescription	111	94.1	88.62, 97.67
Veterinarians	7	5.9	2.63, 11.38
**Places of antibiotics purchase**			
Veterinary pharmacies	74	62.7	53.73, 71.08
Human pharmacies	31	26.3	18.93, 34.76
Drug hawkers	13	11.0	6.27, 17.66
**Dosage determination before usage**			
From accompanied label instructions	51	43.2	34.50, 52.27
Arbitrary	67	56.8	47.73, 65.50
**Administering of antibiotics on fish**			
Self-administered	112	94.9	89.73, 97.91
Veterinary authorities	6	5.1	2.09, 10.27
**Dosage of usage on fish**			
As presented on leaflets	24	20.3	13.80, 28.32
Only a single high dose daily	31	26.3	18.93, 34.76
One single high dose weekly	63	53.4	44.36, 62.25
**Frequently used route for administration**			
Water bath	101	85.6	78.37, 91.09
Infeed	17	14.4	8.91, 21.63
**Withdrawal periods compliance**			
Yes	6	5.1	2.09, 10.27
No	112	94.9	89.73, 97.91
**Reasons for use of antibiotics in aquaculture**			
Bacterial infection treatment	25	21.2	14.52, 29.25
Bacterial infection prevention or prophylaxis	43	36.4	28.13, 45.41
Metaphylaxis	41	34.7	26.57, 43.66
Promotion of growth	9	7.6	3.78, 13.53

CI, confidence interval.

### Pathways for antibiotic residue and resistance spread to humans

Identified significant pathways of antibiotic residue and resistance spread to humans through freshwater fish aquaculture were antibiotic residue contaminated fish and fish products ingestion (*p* = 0.001) and contact with antibiotic residue contaminated fish and fomites (*p* = 0.001) spread. Furthermore, column water and aerosols contaminated with antibiotic residues are pathways for the dissemination of residues to humans from freshwater fish aquaculture environments (*p* < 0.001) (Table 3).

**TABLE 3:** Routes for antibiotic residue and resistance spread from freshwater fish aquaculture to humans in the Southern Guinea Ecological Zone of Northern Nigeria.

Pathway	Low risk		Moderate risk		High risk		Chi-square	*p*
	*n*	%	*n*	%	*n*	%		
**Consumption**	-	-	-	-	-	-	25.06	0.001
Fish with antibiotic residue	33	28.0	70	59.3	15	12.7	-	-
Fish products with antibiotic residue	71	60.2	37	31.3	10	8.5	-	-
**Contacts**	-	-	-	-	-	-	15.38	0.001
Contaminated fish	48	40.7	57	48.3	11	12.7	-	-
Contaminated fomites	78	66.1	33	28.0	7	5.9	-	-
**Environment**	-	-	-	-	-	-	44.57	< 0.001
Contaminated column and wastewater	25	21.2	72	61.1	21	17.8	-	-
Contaminated aerosols from fish aquaculture	75	63.6	37	31.3	6	5.1	-	-

Low risk (< 35%); Moderate risk (35% – 65%); High risk (> 66%); Significant at *p* < 0.05.

### Social and economic activities that drive antibiotic residue and resistance occurrence

Modelled multivariable logistic regression significantly indicated that antibiotic misuse and overuse were more likely to drive antibiotic residue and resistance occurrence in freshwater fish aquaculture (Odds Ratio [OR] = 3.8; 95% confidence interval [CI]: 1.62, 8.74; *p* = 0.002). Also, poor education and expertise of the farmers were more likely to drive antibiotic residues and resistance (OR = 2.9; 95% CI: 1.24, 6.94; *p* = 0.010). The farmers’ poor financial position was five times more likely (OR = 5.1; 95% CI: 1.85, 14.00; *p* = 0.002) to drive residue and resistance occurrence; and the same with the intensive management system that was eight times more likely (OR = 8.5; 95% CI: 3.17, 22.58; *p* = 0.001) to drive the occurrence. Also, poor biosecurity practices and sanitation within and around the farm environment (OR = 5.1; 95% CI: 1.88, 9.31; *p* = 0.001) were more likely to influence antibiotic residues and resistance occurrence in freshwater fish aquaculture (Table 4).

**TABLE 4:** Social and economic factors that drive antibiotic residue and resistance occurrence in freshwater aquaculture fish ponds in the Southern Guinea Ecological Zone of Northern Nigeria.

Factor	Poor driver		Satisfactory driver		Odds ratio (OR)	95% CI	*p*
	*n*	%	*n*	%			
**Antibiotic misuse and overuse**							
Disagree	21	63.6	12	36.4	1.00	-	-
Agree	27	31.8	58	68.2	3.8	1.62, 8.74	0.002
**Poor education and expertise**							
Disagree	17	58.6	12	41.4	1.00	-	-
Agree	29	32.6	60	67.4	2.9	1.24, 6.94	0.010
**Poor financial position of farmers**							
Disagree	11	55.0	9	45.0	1.00	-	-
Agree	19	19.4	79	80.6	5.1	1.85, 14.00	0.002
**Intensive management system**							
Disagree	15	60.0	10	40.0	1.00	-	-
Agree	14	15.1	79	84.9	8.5	3.17, 22.58	0.001
**Poor biosecurity, hygiene, and sanitation of pond environment**							
Disagree	25	61.0	16	39.0	1.00	-	-
Agree	21	27.3	56	72.7	4.2	1.88, 9.31	0.001

CI, confidence interval.

Statistically significant at *p* < 0.05.

### Screening of antibiotic residues with enzyme link immunosorbent assay

The concentrations of TCs residues in fillets ranged from 27.9 μg/kg^−1^ to 31.4 μg/kg^−1^ with a mean value of 29.6 μg/kg^−1;^ in livers, it ranged from 25.6 μg/kg^−1^to 27.9 μg/kg^−1^ with a mean of 26.4 μg/kg^−1^. The TCs residue concentrations in the water column and discharged wastewater ranged from 108.3 μg/kg^−1^ to 133.4 μg/kg^−1^ with 123.6 μg/kg^−1^ mean. The mean values of TCs residue concentrations in fillets, livers and water column and discharged wastewater were: 29.6 ± 9.1 μg/kg^−1^, 26.4 ± 8.6 μg/kg^−1^, and 123.6 ± 18.2 μg/kg^−1^, respectively. However, the mean TCs residue level (123.6 ± 18.2 μg/kg^−1^) above 100 μg/kg^−1^ maximum residue limits (MRL) were observed in the water column and discharged wastewater. The residue concentrations in fillets, livers and column and/or waste were significantly different with mean square = 37597.6 and *p* = 0.001 (Table 5).

**TABLE 5:** Quantification of tetracycline residue concentration in flesh, liver, and column and wastewater samples with enzyme link immunosorbent assay in freshwater fish aquaculture in the Southern Guinea Ecological Zone of Northern Nigeria.

Sample type	Number of positive samples used	Mean ± s.d. (μg/kg^−1^)	95% CI	> MRL
Fillet	6	29.6 ± 9.1	17.35, 36.45	Nil
Liver	6	26.4 ± 8.6	17.38, 35.43	Nil
Column/wastewater	24	123.6 ± 18.2	115.92, 131.29	All

s.d., standard deviation; CI, confidence interval; MRL, maximum residue limit.

### Proportional piles of frequently used antibiotics on ponds

**FIGURE 3:**
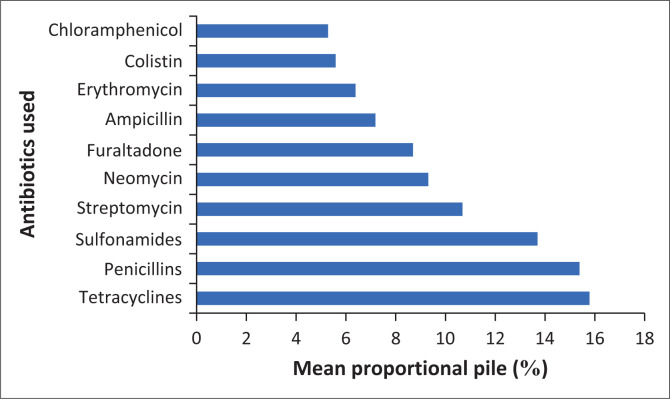
Proportional piling of frequently used antibiotics by fish farmers in the nine fish farming communities of the Southern Guinea Ecological Zone in Northern Nigeria.

Proportional piling was a useful tool extensively used to confirm verbal descriptions in the SSIs. In this study, farmers exhibited adequate existing knowledge about health challenges management in fishing communities. Furthermore, participants averagely ranked commonly applied antibiotics in the fish farms in this order: TCs, penicillin, sulphonamides and streptomycin, among others (Figure 3). The mean proportional pile (relative usage) of TCs among other antibiotics frequently used was 15.8%, which was judged by the farmers as the first most important antimicrobial. There was strong (*W* = 0.979) and significantly concorded statistically (*p* < 0.001) among the fish farmers on the piled antibiotics according to the relative frequencies of use in freshwater fish farms.

## Discussion

The study demonstrates the availability of antibiotic residue in the freshwater water environments of the survey fish farms, which are mostly discharged into rivers and streams that serve as sources of drinking water for humans and animals. This is a consequent public and environmental health challenge that needs to be addressed simultaneously at the human-animal-environment interface. The survey found high proportions of farmers, 78.1% (*n* = 118/151) with a poor knowledge gap about antibiotic use in freshwater fish aquaculture farming in the Southern Guinea Ecological zone of Nigeria. This poor knowledge influences the misuse and overuse of antibiotics in farms. A similar survey in Bangladesh reported fish farmers’ poor knowledge regarding antimicrobial agents’ usage in aquaculture with resultant misuse and overuse (Ali et al. 2014). Bridging the knowledge gap through the implementation of a farmers’ field school approach will create awareness and improve the proper antibiotic use in freshwater aquaculture farming in developing economies.

We found that the majority of the farmers possessed attitudinal behaviour of self-prescription (94.1%, *n* = 111/118) and self-administration (94.9%, *n* = 112/118) of antibiotics in freshwater fish aquaculture without veterinarians’ consultations. This could be associated with the paucity of veterinarians who specialise in aquaculture medical practices in sub-Saharan Africa, especially in Nigeria. The patronage of over-the-counter antibiotics in food of animal origin in Nigeria has been reported to be because of inadequate veterinarians, the high cost of veterinary services and farmers’ poor financial positions (Alhaji et al. 2018). Some farmers acquired antibiotics used in aquaculture from drug hawkers and human pharmacies without prescriptions, thus facilitating misuse and overuse with consequent residues and resistance development. This study found an attitude of non-observance of antibiotics withdrawal periods among farmers. This agrees with the report of non-observance of withdrawal periods on the administered drugs in animals by farmers and consequent residue accumulation in tissues (Alhaji et al. 2021; Vranic et al. 2003).

Farmers used antibiotics to treat and prevent infections and also for growth promotion in freshwater fish aquaculture. This is in agreement with study reports that indicated antibiotics to be used in fish farms as therapeutics, prophylactics and metaphylactics, and also as growth promoters (Cabello 2006; FAO 2005; Grema et al. 2015). The use of antibiotics for metaphylactics or growth promotions in freshwater aquaculture facilitates misuse and overuse, which in turn exert selection pressure on aquatic pathogens, selecting the resistant populations that could contaminate fish and products marketed for human consumption (Cabello 2006; Cabello et al. 2013; Ryu et al. 2012).

The poor biosecurity identified in the surveyed farms, especially the earthen ponds, could be a predisposing factor for the prophylactic and therapeutic uses of antibiotics in them. These often promote antibiotic residue occurrence and the consequent emergence of antibiotic-resistant bacteria (Larissa et al. 2013; Morgan et al. 2011). The attitude of applying low doses of antibiotics in fish farms is noteworthy. Improper administration of antibiotics, especially in low doses, has been reported to facilitate selection bias, driving phenotypic and genetic variability in exposed pathogens, and the consequent emergence of resistant pathogens and genes (Martinez 2008; You & Silbergeld 2014).

We found frequent antibiotic usage in freshwater fish aquaculture to be a common practice. Frequently used agents include TCs, sulphonamides, ampicillin, colistin and chloramphenicol, among others. This has been corroborated by the reports of previous studies that found these antibiotics to be commonly used in aquaculture in developing countries, such as Nigeria (Hassan et al. 2013; Mahmoudi et al. 2014; Olatoye & Basiru 2013). Several classes of antibiotics have been reported to be commonly used in large quantities in the fish industry without regulations, especially in low- and medium-income countries (LMICs), a threat to public and environmental health as well as food safety (Landers et al. 2012; Olatoye & Basiru 2013).

This study found antibiotic residue contaminated fish and fish products ingestion, and residues contaminated fish and fomites contact as significant pathways for dissemination to humans. Furthermore, columns and wastewater, and aerosols contaminated with residues in the freshwater aquaculture environment were found to be significant environmental risk routes for residues to spread to the public. These findings strongly indicate the likelihood of public health risks through water, ingestion of fish, dermal contact, and inhalation of particulate matter, because of the unintended intake of and exposure to antibiotics. Studies have reported consumption of antibiotic-contaminated food animals as well as contact with antibiotic residue contaminated food animals as well as contaminated wastewater spilled into the farm environment as routes for the spread of antibiotic residues, antibiotic-resistant bacteria, and genes (Chang et al. 2014; Marshall & Levy 2011; McEachran et al. 2015; Lhermie, Grohn & Raboisson 2016). Frequent application of antibiotics in aquaculture causes selective pressure on antibiotic-resistant pathogens because they bioaccumulate for a long time in the sediments or water column (Cabello et al. 2016). This can also influence antibiotic resistance genes emergence in fish and the environment (Muziasari et al. 2016).

Social and economic factors significantly drive antibiotic residues and resistance emergence in freshwater fish aquaculture. These include antibiotic misuse and overuse, poor financial positions, poor expertise and education of farmers, poor biosecurity and hygiene, and an intensive husbandry system. Improper use of antibiotics has been reported to significantly drive ABR because of selection pressure on microbiota in food animals (McEwen 2006). Poor biosecurity as well as poor environmental sanitation and hygiene have previously been reported to drive residues and resistance emergence in food of animal origin in developing countries (Alhaji et al. 2019).

The MRL of TCs in fish set by the European Union (EU) countries and the Codex Alimentarius Commission has been 100 μg.kg^−1^ (Canada-Canada, De la Pena & Espinosa-Mansilla 2009; EC 2009; Santos et al. 2016). However, we obtained a concentration of TCs that exceeded the permissible limit in sampled column water and wastewater that mostly drains into the surrounding water bodies. One of the consequences of antibiotic misuse in aquaculture is the bioaccumulation of residues in fish edible tissues and pond environments. Consumption of fish contaminated with antibiotic residue potentially link the aquatic environment and human pathogen’s resistome, a predisposing bridge for antibiotic-resistant bacteria and genes emergence and spread to humans (Cabello et al. 2016). It has been reported that 80% of antibiotics applied on fish aquaculture through feed pass to the water column and sediment, and remain active for a long time at concentrations that facilitate selective pressure on pathogens with consequent development of ABR (Cabello et al. 2016; Samuelsen, Torsvik & Evik 1992; Tamminem et al. 2011).

We found PE tools to be useful in assessing the knowledge and attitudes of farmers on the identification of frequently used antibiotics in aquaculture, the outcomes were very resourceful. The measurements have previously been indicated to be vital in gathering information on diseases and also important for policymaking (Alhaji & Babalobi 2016). It is important to sensitise farmers on the improvement of fish health through adequate biosecurity. Without good sanitation and hygiene of the environment, antibiotics will continue to be used to mitigate infections and disease in ponds, and their misuse and overuse can influence bioaccumulation in sediments for months, creating constant pressure on microbial populations and resistance development (Baquero, Martinez & Canton 2008; Pruden et al. 2013). The detection of antibiotic residues in the collected samples in this research would help in the formulation of appropriate food safety control measures in aquaculture food production.

Multi-discipline collaborations are needed at the grassroots, national, regional and global levels. One Health approach provides the most effective operational intervention for implementation along connecting links as indicated in the conceptual framework. Strong intersectoral collaborations across the human, aquatic, and environmental domains can enhance regular joint surveillance capacity, understanding of the dynamics of AMR emergencies and effective mitigation implementation with the consequent achievement of food safety, and public and environmental health (Alhaji et al. 2021).

This study was limited by its inability to determine the residue concentrations of all antibiotics mentioned by the farmers in the laboratory. This was because of resource constraints brought about by the coronavirus disease 2019 (COVID-19) pandemic. However, the result of TC residue was a significant indicator of residue development in freshwater fish aquaculture in Nigeria. Also, this is a cross-sectional study in which full adjustment for clusters could not be accomplished in the designed probability selection procedure. Nevertheless, this may not have influenced the study’s findings as the farm characteristics and reported antibiotics usage were consistent, and the likely selection bias and imperfection were resolved by the use of confidence intervals.

## Conclusion

The study observed poor knowledge and attitudes towards the use of antibiotics among the surveyed farmers. Farmers’ education about appropriate and best antibiotic practices used in freshwater fish ponds is needed to address current gaps. Also, farmers should be sensitised to adequate biosecurity practices and good hygiene and sanitation practices in farms to reduce infections. High TC residue concentrations detected have critical public and environmental challenges. Collaborations of multi-disciplines and sectors through the ‘One Health’ conceptional framework approach, will mitigate activities that drive residue, resistance occurrence, and consequently assure food safety, food security and public and environmental health. The application of PE techniques concurrently with conventional public and veterinary health procedures is needed for control strategies of antimicrobial mitigation in developing countries and is herewith recommended.
